# An open-label, pragmatic, randomized controlled clinical trial to evaluate the comparative effectiveness of daptomycin versus vancomycin for the treatment of complicated skin and skin structure infection

**DOI:** 10.1186/s12879-015-1261-9

**Published:** 2015-11-07

**Authors:** Teresa L. Kauf, Peggy McKinnon, G. Ralph Corey, John Bedolla, Paul F. Riska, Matthew Sims, Luis Jauregui-Peredo, Bruce Friedman, James D. Hoehns, Renée-Claude Mercier, Julia Garcia-Diaz, Susan K. Brenneman, David Ng, Thomas Lodise

**Affiliations:** Health Economics and Outcomes Research, Merck & Co., Inc., 2000 Galloping Road, Kenilworth, NJ 07033 USA; Global Center for Scientific Affairs, Merck Research Laboratories, Merck & Co., Inc, Kenilworth, NJ USA; Department of Medicine, Duke University Health System, Durham, NC USA; University of Texas at Austin Dell Medical School, Austin, TX USA; Albert Einstein College of Medicine Montefiore Medical Center, Bronx, NY USA; Infectious Diseases Research, William Beaumont Hospital, Royal Oak, MI USA; Medical Research, Mercy Saint Vincent Medical Center, Toledo, OH USA; JM Still Burn Center at Doctors Hospital, Augusta, GA USA; Northeast Iowa Medical Education Foundation, Waterloo, IA USA; Pharmacy and Medicine, University of New Mexico, Albuquerque, NM USA; Department of Infectious Diseases, Ochsner Clinic Foundation, New Orleans, LA USA; Health Economics and Outcomes Research, Optum, Eden Prairie, MN USA; Department of Emergency Medicine, Nassau University Medical Center, East Meadow, NY USA; Albany College of Pharmacy and Health Sciences, Albany, NY USA

**Keywords:** Daptomycin, Vancomycin, Complicated skin and skin structure infection, Pragmatic randomized clinical trial, Antimicrobial, Effectiveness

## Abstract

**Background:**

Treatment of complicated skin and skin structure infection (cSSSI) places a tremendous burden on the health care system. Understanding relative resource utilization associated with different antimicrobials is important for decision making by patients, health care providers, and payers.

**Methods:**

The authors conducted an open-label, pragmatic, randomized (1:1) clinical study (*N* = 250) to compare the effectiveness of daptomycin with that of vancomycin for treatment of patients hospitalized with cSSSI caused by suspected or documented methicillin-resistant *Staphylococcus aureus* infection. The primary study end point was infection-related length of stay (IRLOS). Secondary end points included health care resource utilization, cost, clinical response, and patient-reported outcomes. Patient assessments were performed daily until the end of antibiotic therapy or until hospital discharge, and at 14 days and 30 days after discharge.

**Results:**

No difference was found for IRLOS, total LOS, and total inpatient cost between cohorts. Hospital LOS contributed 85.9 % to the total hospitalization cost, compared with 6.4 % for drug costs. Daptomycin showed a nonsignificant trend toward a higher clinical success rate, compared with vancomycin, at treatment days 2 and 3. In the multivariate analyses, vancomycin was associated with a lower likelihood of day 2 clinical success (odds ratio [OR] = 0.498, 95 % confidence interval [CI], 0.249–0.997; *P* < 0.05).

**Conclusion:**

This study did not provide conclusive evidence of the superiority of one treatment over the other in terms of clinical, economic, or patient outcomes. The data suggest that physician and patient preference, rather than drug acquisition cost, should be the primary driver of initial antibiotic selection for hospitalized patients with cSSSI.

**Trial registration:**

ClinicalTrials.gov: NCT01419184 (Date: August 16, 2011)

**Electronic supplementary material:**

The online version of this article (doi:10.1186/s12879-015-1261-9) contains supplementary material, which is available to authorized users.

## Background

Phase 3, international, multicenter, randomized, double-blind, comparative clinical trials are considered the gold standard for establishing efficacy of antibiotics used for treatment of patients with complicated skin and skin structure infection (cSSSI). Although “efficacy” is established in this study design, it is often difficult to generate quantitative economic or “effectiveness” inferences between treatments in such trials. Making the leap from the comparative efficacy to the comparative effectiveness of an antibiotic used to treat cSSSI is complex and challenging. For example, studies have clearly shown that hospital length of stay (LOS) is the primary driver of the cost of treating patients with cSSSI [1, 2]. However, it is difficult to make direct LOS comparisons from registrational trials because of their structured nature and explicit requirements (i.e., fixed duration of therapy, lack of an oral step-down therapy option during the primary treatment period). Furthermore, evaluating comparative effectiveness requires relaxation of certain parameters included in the design of traditional randomized clinical trials (RCTs), such as patient inclusion and exclusion criteria, monitoring, and restrictions on the use of both the experimental intervention and the comparator agent [3, 4].

Comparative effectiveness evaluations also require the assessment of end points relevant to payers (such as resource utilization and cost) or to patients (such as quality of life, productivity, and the need for follow-up care) that generally are not considered by the U.S. Food and Drug Administration (FDA) as part of the marketing approval process. Likewise, health care providers might be interested in clinical end points that differ from those suggested by FDA guidance documents for a particular therapeutic area. With these considerations in mind, we conducted an open-label, pragmatic RCT (PRCT) to assess the comparative effectiveness of two commonly used medications for cSSSI treatment—daptomycin and vancomycin—with the objective of determining a number of outcomes measures relevant to patients, providers, and payers, such as health cost and clinical success.

## Methods

### Study design and sites

This was a real-world, prospective, open-label, multicenter study conducted from September 2011 through October 2012. The study was conducted at 36 sites (including academic and nonacademic) in the United States. No specific site characteristics, investigator criteria, or prior experience with vancomycin or daptomycin were necessary; however, vancomycin and daptomycin treatment was to be consistent with local practice. All patients provided written informed consent in accordance with local guidelines for studies involving human subjects. The study was conducted in accordance with the 2008 Declaration of Helsinki. Institutional review board (IRB) or ethics committee approval was obtained at each participating center or from a central IRB (Additional file 1). The sections below broadly follow the 10 PRECIS domains proposed by Thorpe et al. [4] to describe aspects of the study that were more pragmatic and more explanatory.

#### Participant eligibility criteria

In keeping with pragmatic trial principles, minimal inclusion criteria were used to identify patients for whom vancomycin or daptomycin would be the initial treatment choice. To be eligible for the study, patients had to be at least 18 years of age and hospitalized for complicated SSSI (cellulitis, cutaneous abscess, wound infection, and burns) caused by suspected or documented methicillin-resistant *Staphylococcus aureus* (MRSA) infection that necessitated intravenous (IV) antibiotics for an anticipated 3 to 14 days of inpatient treatment determined based on physician clinical experience.

Additionally, patients must have had at least three of the following clinical signs and symptoms associated with cSSSI: pain and tenderness with palpation, elevated temperature (>37.5 °C [99.5 °F] oral or >38 °C [100.2 °F] rectal); elevated white blood cell (WBC) count (>10 × 10^9^/L); swelling and/or induration or erythema; or purulent or seropurulent drainage or discharge. Enrollment within 24 h of hospital admission was necessary. Exclusion criteria were similar to those used in phase 3 clinical trials of daptomycin for management of cSSSI [5]; these included presence of bacteremia, osteomyelitis, septic arthritis, or endocarditis at the time of enrollment; requirement for curative surgery (e.g., amputation); and use of systemic antibacterial therapy for the infection for > 24 h within the 48 h preceding the start of administration of study drug, unless the infection Gram-positive pathogen was resistant in vitro to the therapy, or the therapy was administered for 3 or more days with either worsening or no improvement in the infection.

#### Experimental and comparison intervention flexibility

Patients were randomly assigned (1:1) to receive IV vancomycin or IV daptomycin using a computer-generated centralized randomization schedule. Vancomycin is widely used as initial cSSSI treatment and, therefore, was chosen as the comparator [6]. Investigators were advised to administer daptomycin 4 mg/kg once daily, consistent with product labeling [7]. To mirror the range of current clinical practices, vancomycin was dosed at the investigator’s discretion according to institutional protocol. Only the first study drug dose was required by the protocol; all subsequent care decisions were at the discretion of the treating physician and local hospital practice, including when to discontinue treatment, whether to convert from IV therapy to oral therapy, and the length of therapy. No restrictions were imposed regarding concomitant medications, adjunctive procedures, or other therapy.

#### Follow-up intensity

Study assessments were performed at screening; at baseline; daily until the end of cSSSI therapy (including therapy-related adverse event treatment); or at hospital discharge, whichever occurred first. Assessments were also performed at hospital discharge and by telephone interview 14 days and 30 days (±3 days) after hospital discharge. Daily assessments included patient-reported outcomes (described in Trial Secondary End Points), serious adverse events, clinical response, clinician statement of reason for continued hospitalization (if applicable), and study drug dose. Patients receiving vancomycin were also assessed each day to determine whether and when the therapeutic trough level of vancomycin was reached. To reflect the range of current clinical practices, vancomycin levels were obtained and therapeutic drug monitoring was performed at the investigator’s discretion according to institutional protocol.

At discharge, information describing medical resource utilization during hospitalization, including tests, procedures, and number of days in specific wards, was collected from the patient medical record. Follow-up telephone interviews included patient-reported outcomes, continued antibiotic therapy and other medical resource utilization, infection status, and out-of-pocket expenses and time missed from work for patients and caregivers.

#### Trial primary outcome

The primary study end point was infection-related length of stay (IRLOS), because it is objective, easily reproducible, and relevant to the patient, providers, and payers. IRLOS was defined as the number of hours of hospitalization associated with cSSSI management, beginning at study drug initiation and ending at discontinuation of all antibiotic therapy for cSSSI or therapy for antibiotic-related adverse events or at hospital discharge, whichever occurred first. The criteria that determined patient discharge date and time are listed below. However, in order to maintain the pragmatic nature of the trial, the discharge eligibility criteria were not specified in the protocol and it was left to the discretion of the treating physician and local institutional practices.

- If a patient was switched to another treatment after study antibiotic stopped, the IRLOS would continue until the end date of the second medication.
- If the patient experienced an adverse event (AE), the treatment-completion date and time for that AE was recorded as the IRLOS end.
- If the patient finished the treatment before hospital discharge, the end date and time of last dose of vancomycin or daptomycin was the IRLOS end.
- If the patient was still receiving cSSSI treatment at discharge, then the IRLOS would be the same as the total LOS.

#### Analysis of primary outcome

The primary analytic sample (PAS) included all patients with data available to calculate the primary end point (IRLOS). Data were collected in hours and the results were converted to days for ease of interpretation. The PAS excluded patients who discontinued from the study before discharge, those who left the hospital against medical advice, and others for whom a discharge assessment could not be completed.

#### Trial secondary end points

Secondary end points included inpatient medical resource use during initial hospitalization (adjunctive procedures, radiologic and laboratory tests, hospital ward unit), total LOS, cost, and clinical response (cure, improvement, no improvement, or failure). Total length of stay was measured in hours and was analyzed by a fixed-effects model with terms for treatment and the confounding variables. Secondary end points also included the following patient-reported outcomes: pain (as rated by the Brief Pain Inventory [Short Form]) [8]; infection status (“improved a lot,” “improved moderately,” “improved a little,” “no change,” “worsened a little,” “worsened moderately,” or “worsened a lot”) as measured by the Patient Global Impression of Improvement (PGI-I) scale, and quality of life (as rated by the EuroQol 5 Dimensions, 5 Level [EQ-5D-5 L] multiattribute questionnaire) [9].

#### Cost

Costs were estimated from the payer perspective and included direct medical inpatient and outpatient costs. Direct medical costs were based on health resource utilization. To estimate cost of care, unit cost data were obtained from sources external to the study and assigned to corresponding medical resource utilization observed within the trial. Hospital ward costs were applied hourly. General medicine/surgical and intensive care unit costs were estimated via administrative claims using the Optum Research Database (http://www.optum.com/life-sciences/develop-evidence/data-assets.html). Costs for other ward types were not available in the administrative claims data. The average daily cost observed among a similar sample of patients from the Healthcare Cost and Utilization Project (HCUP) [10], adjusted using a payment-to-cost ratio of 1.34 [11], was used as a proxy for other ward costs. The unadjusted average daily cost observed in the HCUP sample was used as a sensitivity analysis.

Vancomycin trough monitoring was estimated using Current Procedural Terminology (CPT) code 80202. Pharmacy, nursing, or other personnel costs related to vancomycin trough monitoring or dose changes were not included in the analysis. For the primary analysis, acquisition costs for study drug and other antibiotics administered during hospitalization were estimated using wholesale acquisition cost (WAC) and attributed the cost of a full vial for partial vial use of daptomycin. Daptomycin was “costed” at $342.31 per 500 mg; vancomycin was costed at $6.13 per gram. As a sensitivity analysis, drug costs were estimated by average wholesale price (AWP) and assumed no waste. (Full results, including outpatient, work loss, and out-of-pocket cost, are available from the authors by request.)

### Statistical methods

#### Sample size

The sample size estimate was based on the primary end point. Based on antibiotic-related LOS from daptomycin clinical trials (data on file) and published literature [12], the expected mean difference in LOS between daptomycin and vancomycin was estimated at approximately 1.5 days (standard deviation [SD], 4.0 days). Assuming equal sample sizes and two-sided significance testing with α = 0.05 and 1 – β = 0.80, it was estimated that 224 patients (112 per group) would be necessary to detect a difference of 1.5 days. A total of 250 patients (125 per group) were to be enrolled to account for expected study attrition of 10 %.

#### Statistical analysis

All descriptive analyses were conducted using the PAS. Descriptive statistics for continuous variables are reported as mean, SD, median, or range, as appropriate. All categorical variables were summarized by frequency and percentage. Patient-reported outcomes of pain and quality of life were assessed as changes from baseline using last observation carried forward methodology. Time to clinical success (cure or improvement) was modeled using Cox proportional hazards. For bivariate analyses, categorical variables were compared using the Fisher exact test, and continuous variables were compared using the Student *t* or Mann–Whitney test.

Stratified analyses were performed to determine whether the infection type and pathogen modified the relationship between treatment and outcomes. Multivariate analyses were performed to quantify the association between treatment and each outcome after adjustment for potential confounding variables. All potential confounding variables (baseline covariates associated with outcomes at *P* < 0.2) were included at model entry and were retained in the model if the associated *P* value was < 0.05. All potential confounding variables were analyzed descriptively and clinically to determine appropriate inclusion in the multivariate analyses of IRLOS, total LOS, total inpatient cost, and clinical success.

Potentially confounding variables included in each model were patient demographics (age, sex, and body mass index [BMI] ≥ 35), presence of a primary care provider (yes/no), baseline Charlson comorbidity score (continuous), baseline blood culture (yes/no), prior all-cause hospitalizations (0, 1, 2, or more), systemic inflammatory response syndrome (SIRS) (count of four conditions), leukocytosis (WBC > 12 × 10^9^/L, yes/no), elevated creatinine level (>132.6 μmol/L, yes/no), infection type, vancomycin use within previous 48 h (yes/no), treatment switch ≥ 24 h preceding discharge (yes/no) or within 24 h of discharge (yes/no), documented MRSA (yes/no), documented Gram-negative infection (yes/no), baseline patient-reported pain score (continuous), patient eagerness for discharge (definitely go home, definitely stay, or unsure), lower extremity infection site (yes/no), cSSSI infection in the preceding 6 months (yes/no), and infectious disease physician as site investigator (yes/no). Multivariate analyses were also controlled for clustering by study site. The continuous outcomes (IRLOS, total LOS, and total inpatient cost) were fitted to a generalized linear model with gamma distribution and log link to account for their skewed distribution. Clinical success was modeled via logistic regression. The multivariate analysis sample (MVAS) included all patients from the PAS with available data on all covariates. All hypothesis testing was performed at the 5 % significance level. Values of test statistics were considered statistically significant when *P* ≤ 0.05.

## Results

### Patients

Of 250 enrolled patients, data were available for 224 (daptomycin, *n* = 118; vancomycin, *n* = 106) to calculate the primary end point of IRLOS; therefore, they comprised the PAS (Table 1). There were no significant differences between the cohorts at baseline; however, a lower percentage of patients was designated as Hispanic/Latino in the daptomycin group than in the vancomycin group (12.7 % vs. 22.6 %, respectively; *P* = 0.05, data not shown). Overall, the patient population included slightly more males than females and was primarily white; mean (SD) BMI was 33.2 (10.3) in daptomycin patients and 34.2 (11.7) in vancomycin patients. Approximately 50 % of the total cohort had been hospitalized during the prior year and 33 % had a previous cSSSI within the preceding 6 months. Fifty percent of patients were receiving vancomycin at the time of enrollment or within 48 h before enrollment. The majority of patients had complicated cellulitis (65.2 %), major cutaneous abscess (40.6 %), or wound infection (15.2 %).

**Table 1:** Baseline demographics and clinical characteristics of the primary analysis sample

Characteristic	Daptomycin (*n* = 118)	Vancomycin (*n* = 106)
Demographics		
Male, *n* (%)	64 (54.2)	57 (53.8)
Age, y, mean (SD)	47.2 (15.2)	50.0 (13.5)
Weight, kg, mean (SD)	97.1 (29.8)	97.4 (30.4)
BMI, mean (SD)	33.2 (10.3)	34.2 (11.7)
Clinical characteristics		
Hospitalizations in preceding year, *n* (%)	61 (51.7)	49 (46.2)
Patients with cSSSI in preceding 6 months, *n* (%)	36 (30.5)	37 (34.9)
Charlson comorbidity score, mean (SD)	1.1 (1.6)	1.1 (1.3)
Patients with diabetes, *n* (%)	34 (28.8)	37 (34.9)
SIRS symptoms, *n* (%)		
Temperature > 38 °C or > 100.4 °F	19 (16.1)	12 (11.3)
Heart rate > 90 beats/minute	54 (45.8)	43 (40.6)
Tachypnea (> 20 breaths/minute)	12 (10.2)	6 (5.7)
Blood pressure < 90/50 mmHg	5 (4.2)	1 (0.9)
WBC count > 12 × 10^9^/L	61 (51.7)	41 (38.7)
Blood urea nitrogen > 8.9 mmol/L	12 (10.2)	6 (5.7)
Creatinine > 132.6 μmol/L	12 (10.2)	9 (8.5)
Patient-reported pain score, mean (SD)	6.6 (3.0)	6.9 (3.2)
Vancomycin use at enrollment or within 48 h before randomization, *n* (%)	65 (55.1)	50 (47.1)
Baseline blood culture, *n* (%)	82 (69.5)	76 (71.7)
Gram-negative infection, *n* (%)^a^	11 (16.7)	7 (10.8)
cSSSI diagnosis, *n* (%)		
Complicated cellulitis^b^	81 (68.6)	65 (61.3)
Major cutaneous abscess	45 (38.1)	46 (43.4)
Wound infection	18 (15.3)	16 (15.1)
Erysipelas	0 (0)	0 (0)
Diabetic ulcer	2 (1.7)	5 (4.7)
Nondiabetic ulcer (stasis ulcer/decubitus ulcer)	2 (1.7)	3 (2.8)
Bite wound	1 (0.9)	1 (0.9)
Burn wound	0 (0)	0 (0)
Other^c^	2 (1.7)	3 (2.8)

*BMI* body mass index, *cSSSI* complicated skin and skin structure infection, *SD* standard deviation, *SIRS* systemic inflammatory response syndrome, *WBC* white blood cell

^a^Based on the number of patients with primary skin infection lesion culture obtained at baseline (daptomycin, *n* = 66; vancomycin, *n* = 65)

^b^Defined as cellulitis that requires hospitalization and treatment with IV antibiotics suspected or documented to be caused by MRSA

^c^“Other” infection sites include groin, axillae, breast, back, suprapubic region, genitalia, anterior perineum, face, hand, and preseptal region

Baseline data were incomplete for three patients in the daptomycin group. Therefore, the MVAS consisted of 115 patients in the daptomycin group and 106 patients in the vancomycin group. After hospital discharge, 183 patients (daptomycin, *n* = 92; vancomycin, *n* = 91) responded to the 14-day interview and 196 patients (daptomycin, *n* = 101; vancomycin, *n* = 95) responded to the 30-day interview.

### Health economic outcomes

For the primary end point of IRLOS, there was no significant difference between the daptomycin and vancomycin arms for either PAS or MVAS (Table 2). The LOS in one (0.9 %) daptomycin and two (1.9 %) vancomycin patients extended beyond management of cSSSI because of treatment of adverse events that resulted from treatment of the cSSSI. In addition, neither total LOS nor total inpatient cost was significantly different between cohorts under unadjusted or adjusted conditions. In our base case analysis, assuming WAC and waste of partial daptomycin vials, mean total inpatient cost was $9641 for the daptomycin arm and $9083 for the vancomycin arm. Hospital LOS contributed 85.9 % to the total hospitalization cost, compared with 6.4 % for drug costs.

**Table 2:** Health economic outcomes

Outcome	Unadjusted (PAS)			Adjusted (MVAS)	
	Daptomycin (*n* = 118)	Vancomycin (*n* = 106)	*P* value	Rate ratio^b^	*P* value
IRLOS, hours, mean (SD)	91.5 (57.8)	93.2 (60.8)	0.823	1.002 (0.844–1.191)	0.979
Total LOS, hours, mean (SD)	98.5 (77.0)	101.2 (72.1)	0.785	1.018 (0.861–1.204)	0.833
Total inpatient cost, 2012 US$, mean (SD)^a^	9641 (6683)	9083 (5855)	0.509	0.940 (0.803–1.101)	0.442

*IRLOS* infection-related length of stay, *LOS* length of stay, *MVAS* multivariate analytic sample, *PAS* primary analytic sample, *SD* standard deviation

^a^Wholesale acquisition cost estimates using “waste” algorithm

^b^Rate ratio reflects effect of vancomycin relative to daptomycin on outcome of interest. Ratios reported as mean (95 % confidence interval)

**Fig. 1:**
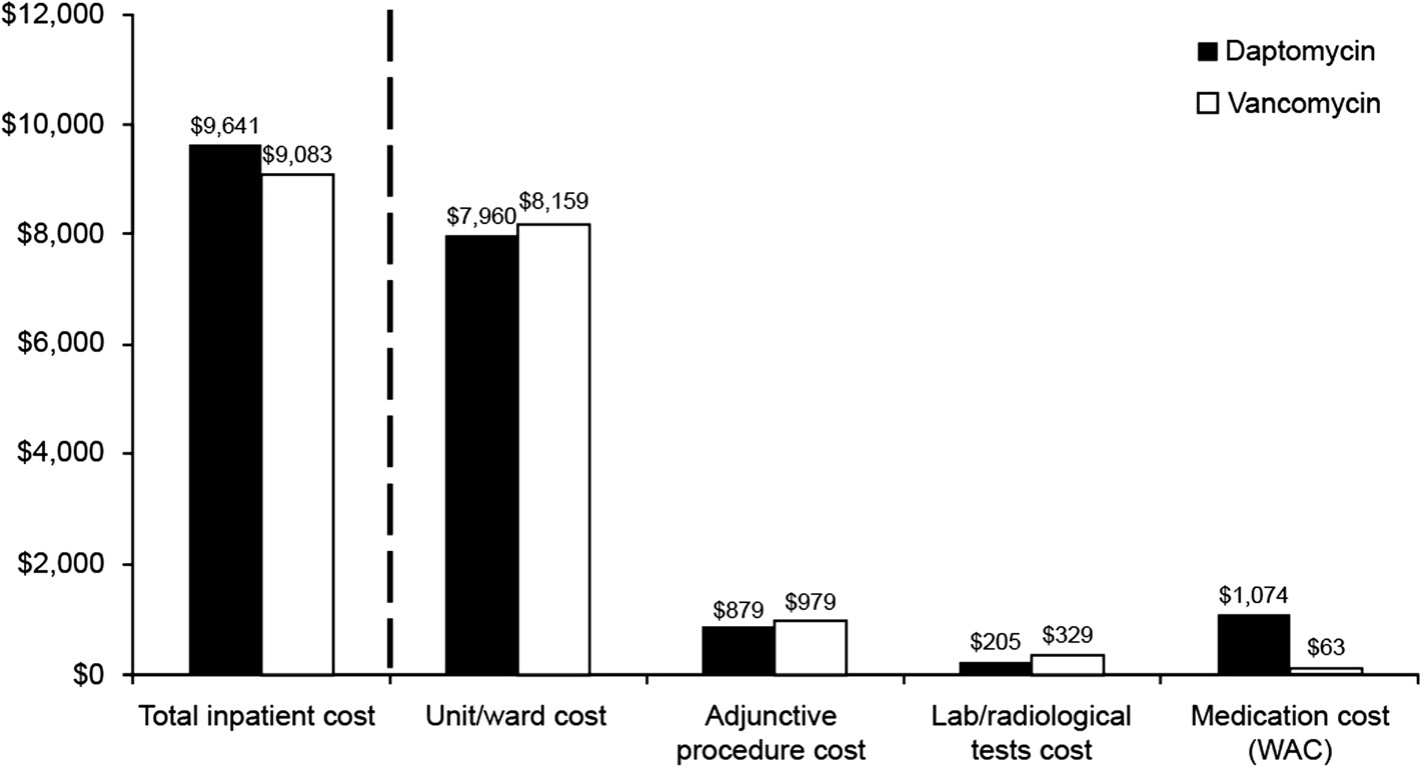
Mean inpatient costs by components. Drug cost calculation assumes waste of partial daptomycin vials. *P* = NS for all. Ward/unit cost was defined as the cost care in a particular unit or ward. Adjunctive procedures included incision/drainage, surgical debridement/excision, amputation, device removal/replacement, wound specialist services, and physical therapy. Radiology tests included radiography, medical resonance imaging, ultrasound, and computed tomography. WAC, wholesale acquisition cost

In the sensitivity analysis, in which AWP was used and there was no assumed drug wasted, the mean total inpatient cost was $9409 for daptomycin and $9106 for vancomycin. Hospital LOS was the primary driver of cost in both treatment arms (Fig. 1). The drug cost for daptomycin was significantly greater than that of vancomycin. Conversely, daptomycin was associated with lower laboratory and radiologic test costs than vancomycin (*P* < 0.001 for both comparisons; Fig. 1). No statistically significant difference in IRLOS, LOS, and total inpatient cost between the daptomycin and vancomycin cohorts was seen when these parameters were analyzed by infection type or pathogen (Table 3).

**Table 3:** Health economic outcomes by infection type and pathogen (unadjusted)^a–c^

	Abscess		Cellulitis		Wound	
By infection type^d^	Daptomycin	Vancomycin	Daptomycin	Vancomycin	Daptomycin	Vancomycin
	(*n* = 40)	(*n* = 42)	(*n* = 54)	(*n* = 44)	(*n* = 17)	(*n* = 16)
IRLOS, hours, mean (SD)	83.8 (53.8)	102.1 (75.4)	97.3 (61.5)	80.2 (43.5)	94.4 (63.4)	109.0 (59.0)
Total LOS, hours, mean (SD)	94.8 (102.2)	105.6 (75.2)	101.7 (64.4)	85.1 (56.6)	103.0 (58.9)	139.3 (94.3)
Total inpatient cost, 2012 US$, mean (SD)^a^	9515 (8501)	9432 (5955)	9871 (5805)	7415 (4750)	9814 (5927)	13,101 (7025)
	*Staphylococcus aureus* ^e^		MRSA		MSSA	
By pathogen	Daptomycin	Vancomycin	Daptomycin	Vancomycin	Daptomycin	Vancomycin
	(*n* = 58)	(*n* = 43)	(*n* = 39)	(*n* = 27)	(*n* = 14)	(*n* = 10)
IRLOS, hours, mean (SD)	98.4 (64.5)	101.2 (69.4)	98.5 (67.0)	85.9 (51.8)	84.5 (43.6)	136.2 (95.5)
Total LOS, hours, mean (SD)	109.0 (95.9)	105.4 (69.3)	111.3 (108.5)	92.3 (53.7)	93.0 (54.2)	136.2 (95.5)
Total inpatient cost, 2012 US$, mean (SD)^a^	10,508 (8109)	9604 (5691)	10,692 (9171)	8854 (4119)	8967 (4084)	11,308 (8345)

*IRLOS* infection-related length of stay, *LOS* length of stay, *MRSA* methicillin-resistant *Staphylococcus aureus*, *MSSA* methicillin-susceptible *Staphylococcus aureus*, *SD* standard deviation, *WAC* wholesale acquisition cost

^a^Multivariate analysis sample of 115 daptomycin and 106 vancomycin patients

^b^WAC cost estimates using “waste” algorithm

^c^The *P* value was nonsignificant for all comparisons between daptomycin and vancomycin

^d^Four daptomycin and four vancomycin patients had other infection types

^e^Includes known *S. aureus* infection for those patients with a culture; please note 5 daptomycin and 6 vancomycin patients were dropped from the MRSA/MSSA subgroup analyses mostly due to missing data

### Clinical success and patient-reported outcomes

**Fig. 2:**
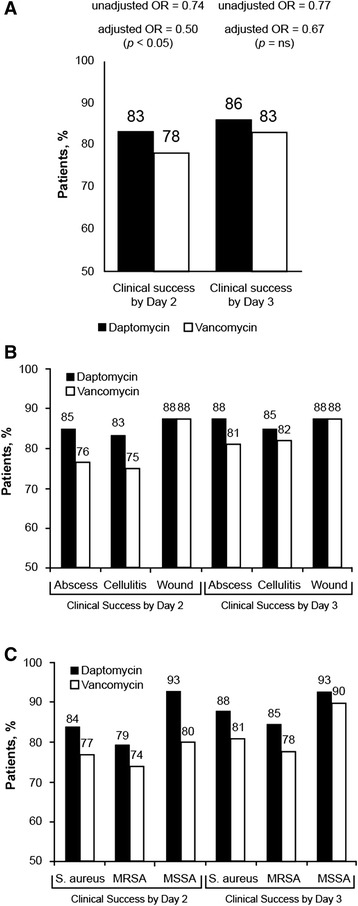
Proportion of patients achieving clinical success by day 2 and day 3: overall (**a**), by infection type (**b**), and by pathogen (**c**). Clinical success was defined as improvement or cure. **a** Odds ratio (OR) for vancomycin compared with daptomycin. **b** Clinical success rates by infection type, excluding the 4 daptomycin and 4 vancomycin patients with “other” infection types. **c** Clinical success rates by pathogen, includr known *S. aureus* infection for those patients with a culture. MRSA, methicillin-resistant *Staphylococcus aureus*; MSSA, methicillin-susceptible *Staphylococcus aureus.* See Table 3 for sample sizes

Because all patients reached clinical success (i.e., improvement or cure) by the end of their inpatient stay, clinical success was defined for analysis purposes as success within 2 to 3 days of randomization. A greater proportion of daptomycin-treated than vancomycin-treated patients achieved clinical success by day 2 and day 3 (Fig. 2a). Although the unadjusted differences in clinical success were not significant, logistic regression analysis showed that vancomycin treatment, relative to daptomycin treatment, was associated with a decreased chance of achieving clinical success within 2 days (odds ratio [OR] = 0.498; 95 % confidence interval [CI], 0.249–0.997; *P* = 0.049). Significant variables in the 2-day response included count of SIRS (*P* = 0.041), Gram-negative infection (*P* = 0.006), and baseline vancomycin use (*P* = 0.031). Similarly, clinical success rates were not significantly different within 2 and 3 days of treatment when analyzed by infection type (Fig. 2b) or pathogen (Fig. 2c).

No notable differences in patient-reported outcomes (pain, health-related quality of life, or infection status) by group were observed. Patient assessments of infection improvement mirrored clinical assessments: nearly 80 % of patients reported “a little,” “moderately,” or “a lot” improved by day 2. Patient-reported quality of life and pain scores also showed rapid improvement in both treatment groups.

## Discussion

The purpose of this trial was to examine therapy in patients deemed by the investigator to need IV anti-MRSA antibiotic treatment. Patients whom the investigator decided could be treated with an oral agent would not fall within the inclusion criteria for this study. In addition, this study was not designed to examine if inpatient length of stay could be reduced by the administration of oral agents (i.e., trimethoprim/sulfamethoxazole or clindamycin), although switching to other antibiotic was accounted for in the IRLOS calculation.

Overall, we observed comparable and relatively short IRLOS and total LOS across study groups, which translated into similar total inpatient costs. In our study, LOS contributed 85.9 % to the total cost of hospitalization, compared with 6.4 % for drug costs. From an administrative perspective, there might be a tendency for institutions to weigh drug acquisition cost as a major determinant of formulary inclusion once safety and efficacy concerns are met. This is understandable because pharmacy expenditure is a highly visible budget component. However, overall inpatient costs in this study were equivalent, despite large differences in acquisition costs between the two drugs. This finding highlights the importance of LOS rather than drug cost as the primary component of total inpatient cost in cSSSI.

Although we did not find significant differences in IRLOS or total inpatient cost, we observed significantly greater odds of achieving clinical success by day 2 in the daptomycin group than in the vancomycin group and positive trends in the daptomycin group for key patient subgroups based on infection site and pathogen. This finding might be due to a potential for partial crossover in patients who received vancomycin for less than 24 h, though an analysis of factors contributing to the early clinical response was not included in the study design. By day 3, more than 80 % of patients in both groups achieved clinical success, yet average LOS was about 4 days whether patients achieved clinical success by day 2 or day 3. The unblinded study design may also have impacted the assessment of clinical response, and is a limitation of the study. Our findings suggest an opportunity to further decrease LOS, which is the primary driver of health care costs for patients with cSSSI. One approach would be to speed the transition to outpatient treatment for patients who are responding and who do not require treatment in the hospital. The optimal discharge plan is likely to vary widely depending on hospital, patient, and environmental factors but could include a form of outpatient parenteral antibiotic therapy for certain patients who show early clinical success. A focus on facilitating appropriate discharge based on clinical response should have a positive benefit on overall health care use, even if resulting LOS reductions are modest.

The economic outcomes of this study are subject to several limitations that should be considered when interpreting the results. First, because cultures were not obtained for all patients, we cannot rule out an imbalance in methicillin-susceptible *Staphylococcus aureus* (MSSA) that favors the daptomycin arm, which might have affected our findings because vancomycin has been associated with increased risk for treatment failure in MSSA infections compared with other antibiotics [13]. In addition, we relied largely on an administrative database of commercial health plans to estimate hospital costs. Such costs naturally will vary by hospital. Another potential limitation of the study is that drug acquisition costs are expected to vary by institution. We used the WAC for the base case analysis as our best estimate of the cost that most hospitals pay for inpatient medication. Use of the AWP increased the total drug cost in both groups, particularly the daptomycin group. Both WAC- and AWP-based cost estimates were subject to two methods of daptomycin costing. In the base case, we assumed that partial doses of daptomycin were wasted (e.g., a patient needing 600 mg of daptomycin would be assigned two vials for costing purposes). A less conservative approach assumed no wasting of daptomycin, which decreased the difference in estimated cost compared with that of vancomycin. Combining the unit cost options and the possible waste provided best-case (WAC, no waste) and worst-case (AWP, with waste) estimates of additional inpatient costs attributable to daptomycin of $235 and $665, respectively.

In the current study, patients in the vancomycin cohort were more likely than those in the daptomycin cohort to have a dose adjustment. The cost of dose adjustments in terms of nursing or pharmacy time or consultation were not included in the analysis; only the nursing time and laboratory cost associated with trough measurement were included. Many institutions use a pharmacy consultant or other service to monitor vancomycin patients, and daptomycin use might result in a cost offset because of the lack of a requirement for therapeutic drug monitoring [14, 15]. Costs or outcomes associated with antibiotic stewardship or the development of resistance were not assessed in this study.

Finally, the current study was not powered to detect differences in cost, and none of the cost outcomes differed statistically between the two study groups, with the exception of drug and laboratory costs. The premise for a 1.5-day difference in LOS was based on previous daptomycin clinical trial data and single-center studies that reported an average LOS of approximately 4 to 8 days (data on file, Merck & Co., Inc.) [12]. In the intervening period, LOS for cSSSI has steadily decreased [10]. Although the intent of our study was to enroll patients with an anticipated LOS of at least 3 days, only 54.9 and 58.0 % of patients met that criterion *ex post facto* for IRLOS and total LOS, respectively.

## Conclusions

This pragmatic clinical trial of daptomycin compared with vancomycin for management of cSSSI caused by suspected or documented MRSA, similar to many PRCTs [16], did not provide conclusive evidence of the superiority of one treatment over the other in terms of clinical, patient, or economic outcomes. However, it did identify the potential for continued reduction in LOS with more rapid discharge of patients who showed clinical improvement, an idea that deserves further study. The trial also highlights the importance of evaluating the total cost of inpatient care rather than focusing solely on drug acquisition cost. In summary, the data suggest that health care provider and patient preference, rather than drug acquisition cost, should be the primary driver of initial antibiotic selection for hospitalized patients with cSSSI. Physician and patient preferences can be driven by many factors, including but not limited to physician experience, dosing frequency, laboratory monitoring, and the potential for earlier discharge.

## Additional file

Additional file 1:
**Daptomycin Pragmatic Skin Trial IRB and Participating Center List.** (DOCX 18 kb)

## Abbreviations

AE: Adverse event
AWP: Average wholesale price
BMI: Body mass index
CI: Confidence Interval
CPT: Current Procedural Terminology
cSSSI: Complicated skin and skin structure infection
EQ-5D-5 L: EuroQol 5 Dimensions, 5 Level
FDA: Food and Drug Administration
HCUP: Healthcare Cost and Utilization Project
IRB: Institutional review board
IRLOS: Infection-related length of stay
IV: Intravenous
LOS: Length of stay
MRSA: Methicillin-resistant *Staphylococcus aureus*
MSSA: Methicillin-susceptible *Staphylococcus aureus*
MVAS: Multivariate analysis sample
OR: Odds ratio
PAS: Primary analytic sample
PGI-I: Patient Global Impression of Improvement
PRCT: Pragmatic randomized clinical trial
RCTs: Randomized clinical trials
SD: Standard deviation
SIRS: Systemic inflammatory response syndrome
WAC: Wholesale acquisition cost
WBC: White blood cell
