# Supplementary material for: An open-label, pragmatic, randomized controlled clinical trial to evaluate the comparative effectiveness of daptomycin versus vancomycin for the treatment of complicated skin and skin structure infection
Source: BMC Infect Dis. 2015 Nov 7;15:503. doi: 10.1186/s12879-015-1261-9 (PMC4637139; doi:10.1186/s12879-015-1261-9)
Supplement: Additional file 1: — Daptomycin Pragmatic Skin Trial IRB and Participating Center List. (DOCX 18 kb) [file 12879_2015_1261_MOESM1_ESM.docx]

|  |  |
| --- | --- |
| **Institutional Review Board** | **Participating Center** |
| Western Institutional Review Board (WIRB) | Regional Health Clinical Research |
| North Memorial Medical Center/North Memorial Health | Infectious Diseases MPLS-LTD |
| St. Vincent Mercy Med Center IRB | ID Clinical Research, Ltd. |
| Western Institutional Review Board (WIRB) | Upstate Infectious Disease Associates, LLP |
| Reliant Medical Group, Inc. | Saint Vincent Hospital at Worcester Medical Center |
| Winthrop-University Hospital | Winthrop University Hospital Clinical Trials Center |
| Ochsner Clinic Foundation | OCHSNER Clinic Foundation |
| Henry Ford Health System IRB | Henry Ford Health System |
| Biomedical Research Alliance New York (BRANY) | Montefiore Medical Center |
| Palomar IRC | eStudySite, Escondido |
| Sharp IRB | eStudySite, Chula Vista |
| Sharp IRB | eStudySite, LaMesa |
| Sunrise Health IRB | eStudySite, Las Vegas |
| Western Institutional Review Board (WIRB) | eStudySite, Oceanside |
| Western Institutional Review Board (WIRB) | Hospital Physicians in Clinical Research, PLLC |
| Forsyth Mem Hosp, Inc. IRB #1 - Biomedical | Novant Clinical Research Institute |
| Western Institutional Review Board (WIRB) | Horizon Research Group, Inc. |
| Human Investigation Committee | William Beaumont Hospital |
| DeKalb Medical IRB | Atlanta Institute for Medical Research |
| University of Louisville IRB | University of Louisville |
| Western Institutional Review Board (WIRB) | Michigan State University |
| OhioHealth Institutional Review Board | Remington-Davis, Inc. |
| Western Institutional Review Board (WIRB) | Temple University School of Medicine |
| Dignity Health IRB | HealthCare Partners Medical Group |
| Western Institutional Review Board (WIRB) | Joseph M Still Research Foundation, Inc. |
| Biomedical Research Alliance New York (BRANY) | Nassau University Medical Center |
| Palmetto Health IRB | Three Rivers Medical Associates |
| University of New Mexico Health Sciences Center IRB | Medical University of South Carolina |
| Human Research Protection Office | University of New Mexico |
| Western Institutional Review Board (WIRB) | HealthCare Partners Medical Group |
| National Center for Child Health and Development I | Louisiana State University Health Sciences Center |
| Western Institutional Review Board (WIRB) | Prevention & Strengthening Solutions, Inc |
| Western Institutional Review Board (WIRB) | Cotton-O'Neil Clinical Research Center |
| Western Institutional Review Board (WIRB) | Endocrine Lipid Diabetes Research Institute |
| Wheaton Franciscan Healthcare IRB | Northeast Iowa Medical Education Foundation |
| Committee on Human Research (CHR) | San Francisco General Hospital |
